# Intelligent Optimization Design of a Phononic Crystal Air-Coupled Ultrasound Transducer

**DOI:** 10.3390/ma16175812

**Published:** 2023-08-24

**Authors:** Jianghai Wang, Huawei Ji, Anqi Qi, Yu Liu, Liming Lin, Xin Wu, Jing Ni

**Affiliations:** School of Mechanical Engineering, Hangzhou Dianzi University, Hangzhou 310018, China212010113@hdu.edu.cn (Y.L.);

**Keywords:** phononic crystal air-coupled ultrasonic transducer, radial basis function neural network, NSGA-II algorithm, −6 dB bandwidth

## Abstract

To further improve the operational performance of a phononic crystal air-coupled ultrasonic transducer while reducing the number of simulations, an intelligent optimization design strategy is proposed by combining finite element simulation analysis and artificial intelligence (AI) methods. In the proposed strategy, the structural design parameters of 1–3 piezoelectric composites and acoustic impedance gradient matching layer are sampled using the optimal Latin hypercube sampling (OLHS) method. Moreover, the COMSOL software is utilized to calculate the performance parameters of the transducer. Based on the simulation data, a radial basis function neural network (RBFNN) model is trained to establish the relationship between the design parameters and the performance parameters. The accuracy of the approximation model is verified through linear regression plots and statistical methods. Finally, the NSGA-II algorithm is used to determine the design parameters of the transducer. After optimization, the band gap widths of the piezoelectric composites and acoustic impedance gradient matching layer are increased by 16 kHz and 13.5 kHz, respectively. Additionally, the −6 dB bandwidth of the transducer is expanded by 11.5%. The simulation results and experimental results are consistent with the design objectives, which confirms the effectiveness of the design strategy. This work provides a feasible strategy for the design of high-performance air-coupled ultrasonic transducers, which is of great significance for the development of non-destructive testing technology.

## 1. Introduction

Due to its high precision, high resolution, safety, and non-destructive, air-coupled ultrasonic detection technology has been widely used in industrial inspections [1,2,3,4]. The transducer is the core component of the air-coupled ultrasonic detection system, and its performance determines the accuracy and efficiency of the system. The traditional air-coupled ultrasonic transducer is prone to experiencing coupling between thickness vibrations and lateral vibrations under high sound intensity and high-power conditions [5,6]. Additionally, due to the significant difference in acoustic impedance between the piezoelectric material and air, strong reflections occur at the interface [7], which hinders the transducer from achieving the expected performance and meeting the requirements of modern air-coupled ultrasonic inspection. Therefore, designing and optimizing high-performance ultrasonic transducers is of paramount importance.

In recent years, efforts have been made to enhance the performance of ultrasonic transducers in two aspects. On one hand, researchers have focused on suppressing lateral vibrations by employing various methods, such as adding decoupling materials to polymers [5,6,8], using air as a substitute for polymers, and applying for metal plates on ceramic column surfaces [9,10]. On the other hand, acoustic impedance gradient matching techniques have gained attention. Compared to traditional quarter-wavelength single/multi-layer matching techniques, acoustic impedance gradient matching effectively broadens the transducer’s operating bandwidth and improves sensitivity, enhancing overall performance [11,12,13]. Phononic crystal, with its characteristics such as bandgaps and defective state, offers control over the propagation of sound and elastic waves [14,15,16]. Ji et al. [17,18] designed a phononic crystal air-coupled ultrasonic transducer. By incorporating the phononic crystal structure into the 1–3 piezoelectric composites and the acoustic impedance gradient matching layer, they effectively suppressed lateral vibration coupling during the transducer’s operation. Furthermore, they achieved good impedance matching, thereby expanding the transducer’s operating bandwidth. However, due to the complexity of the structural parameters involved, traditional design methods cannot directly obtain the optimal parameters.

Artificial intelligence (AI) has been widely used to solve complex engineering modeling and optimization problems due to its unique advantages [19,20,21]. Radial basis function neural networks (RBFNN) [22], as a common AI method, are widely employed in areas such as image recognition [23,24], nonlinear control [25,26], and structural optimization [27], thanks to their concise training approach, good convergence properties, and strong ability to approximate nonlinear functions. Furthermore, NSGA-II [28,29,30,31] is one of the most popular multi-objective genetic algorithms, which reduces the complexity of non-dominated sorting genetic algorithms and offers fast execution speed and good convergence of solution sets. Considering these excellent modeling and optimization capabilities, AI methods can be effectively applied to the optimization design of phononic crystal air-coupled ultrasonic transducers, thereby improving their operational performance.

In this paper, an optimization design strategy for a phononic crystal air-coupled ultrasonic transducer is proposed based on AI methods, which further improves the working performance of a phononic crystal air-coupled transducer while reducing the number of simulations. Firstly, the optimal Latin hypercube sampling (OLHS) method is used to sample the design parameters of the 1–3 piezoelectric composites, including the thickness and radius of the piezoelectric columns, as well as the thickness and period of the acoustic impedance gradient matching layer. Then, the COMSOL software is employed to calculate performance parameters such as the upper and lower limits of the bandgaps in the piezoelectric composite, the upper and lower limits of the bandgaps in the acoustic impedance gradient matching layer, the operating frequency and the −6 dB operational bandwidth of the ultrasonic transducer. Based on the simulation data, a RBFNN model is trained to describe the relationship between the design parameters and performance parameters. The accuracy of the approximate model is validated through error analysis using linear regression plots and statistical methods. Subsequently, the NSGA-II optimization algorithm is utilized to obtain the optimal design parameters. Finally, the optimized transducer is subjected to simulation and experimental validation, confirming the effectiveness of the proposed optimization design strategy.

## 2. Phononic Crystal Air-Coupled Ultrasound Transducer Model

### 2.1. 1–3 Piezoelectric Composites Model

**Figure 1:**
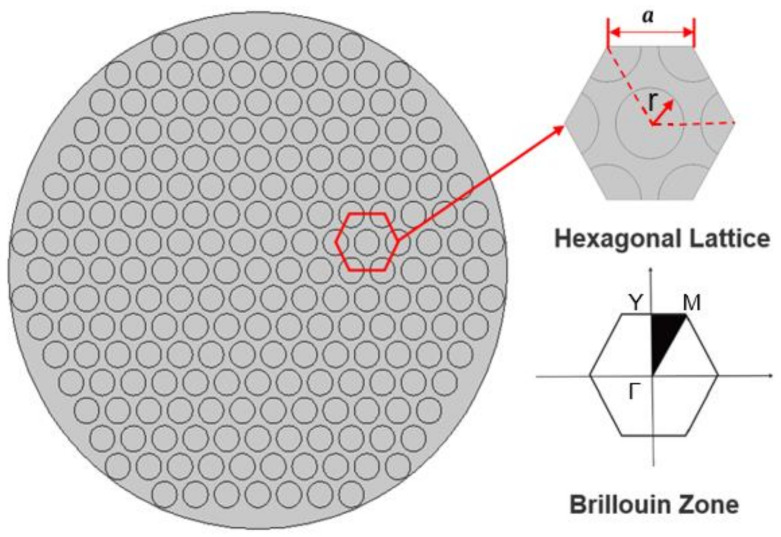
Hexagonal lattice piezoelectric composite.

The hexagonal lattice arrangement of piezoelectric columns can better suppress lateral vibrations in 1–3 piezoelectric composites, resulting in improved performance [17]. Therefore, in this study, the hexagonal lattice piezoelectric composite (Figure 1) is employed, with the following structural parameters: radius R = 20 mm, thickness T, lattice constant a = 2.5 mm, and piezoelectric column radius r. The piezoelectric phase material is PZT-5A, polarized along the thickness direction, and the polymer phase material is epoxy resin, with specific parameters listed in Table 1.

### 2.2. Acoustic Impedance Gradient Matching Layer Model

**Figure 2:**
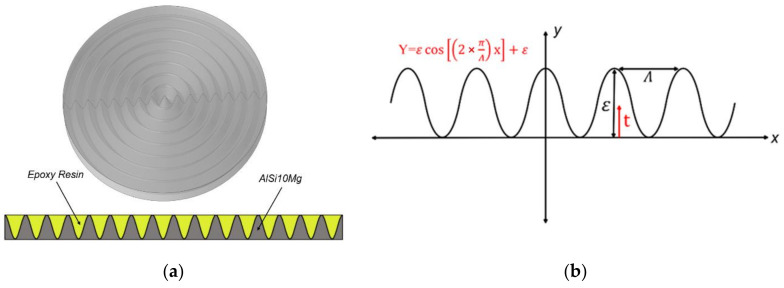
Phononic crystal acoustic impedance gradient matching layer: (**a**) 3D model and (**b**) Cross-sectional function.

In order to effectively suppress lateral vibrations of the transducer while achieving good acoustic impedance matching, Ji et al. [18] designed a phononic crystal acoustic impedance gradient matching layer (Figure 2) composed of two materials with high and low acoustic impedance. The material near the piezoelectric material end is AlSi10Mg, and the material near the transmission medium end is epoxy resin. The specific parameters of the materials are listed in Table 2. The intersection line equation between the two materials (Figure 2b) is considered a function:

(1) $$Y=\varepsilon \cos\left[\left(2\times \frac{\pi }{\Lambda }\right)x\right]+\varepsilon$$

where *ε* represents the thickness of the acoustic impedance gradient matching layer, and the acoustic impedance of the matching layer changes with the variation of the parameter t (0 ≤ t ≤ *ε*) in the thickness direction, and Λ represents the period of the intersection line equation function, which is the lattice constant of phononic crystal.

## 3. Optimization Design Strategy for Phononic Crystal Air-Coupled Ultrasound Transducer

Based on simulation data, an optimization design strategy for the piezoelectric layer and acoustic impedance gradient matching layer of phononic crystal air-coupled ultrasonic transducer is proposed. The design parameters of the transducer include the thickness of the 1–3 piezoelectric composites, the radius of the piezoelectric column, the thickness, and the period of the acoustic impedance gradient matching layer. The performance parameters include the upper and lower limits of the bandgap of the 1–3 piezoelectric composites, the upper and lower limits of the bandgap of the acoustic impedance gradient matching layer, the operating frequency, and −6 dB bandwidth of the ultrasonic transducer. In the optimization design strategy, the OLHS method is first used to sample the data. Then, RBFNN is employed to model the nonlinear relationship between the design parameters and performance parameters. Error analysis is conducted to validate the reliability of the obtained approximation model. Finally, the NSGA-II genetic algorithm is utilized to determine the optimal design parameters.

### 3.1. Radial Basis Function Neural Network Model

#### 3.1.1. Approximate Model Construction

Structural optimization of phononic crystal air-coupled ultrasound transducer is achieved through numerical optimization methods. To begin with, it is necessary to establish the functional relationship between the output responses and the structural parameters. However, in phononic crystal theory, particularly in solid/solid phononic crystal, elastic waves undergo complex reflection, transmission, and scattering phenomena at the interfaces of different materials. These various effects are coupled together, making the mechanism of bandgap formation more intricate. Currently, there is no precise theory available to accurately describe the relationship between structural parameters and bandgap frequencies. Furthermore, the highly complex nonlinear mapping relationship between the structural parameters and performance parameters of the transducer cannot be obtained through theoretical calculations alone. Therefore, in the structural optimization design, we employ an approximate model to establish the relationship between the input sample data and the output responses.

**Figure 3:**
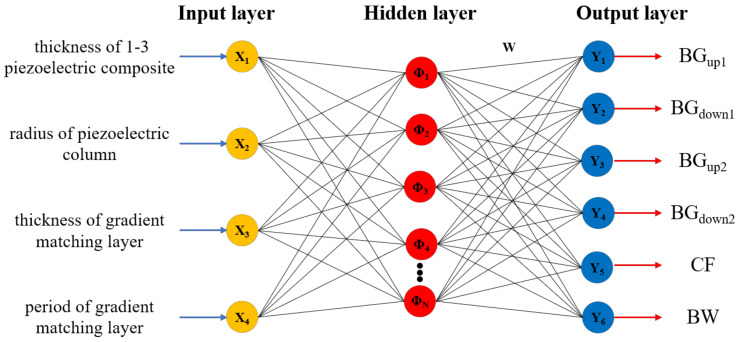
RBFNN model.

In this paper, the RBFNN model is chosen as the approximate model in the optimization of phononic crystal air-coupled ultrasonic transducer. The RBFNN typically consists of an input layer, a hidden layer, and an output layer, as shown in Figure 3. Taking into consideration the impact of various structural parameters on the performance of phononic crystal air-coupled ultrasonic transducer, this study selects four parameters: the thickness of 1–3 piezoelectric composites, the radius of the piezoelectric column, the thickness of the acoustic impedance gradient matching layer, and the period of the matching layer as the input variables for optimization. These parameters are denoted as X_1_, X_2_, X_3_, and X_4_ in the experimental design. The initial values and ranges of these parameters are shown in Table 3. When the operating frequency of the phononic crystal air-coupled ultrasound transducer is within the bandgaps of both the piezoelectric composites and the acoustic impedance gradient matching layer, it exhibits optimal suppression of lateral vibrations [17]. The −6 dB bandwidth of the ultrasonic transducer represents its higher axial resolution and serves as an important indicator of its performance. Therefore, in this study, the upper limits of the bandgap of 1–3 piezoelectric composites (BG_up1_), the lower limits of the bandgap of 1–3 piezoelectric composites (BG_down1_), the upper of the bandgap of the acoustic impedance gradient matching layer (BG_up2_), the lower limits of the bandgap of the acoustic impedance gradient matching layer (BG_down2_), the center frequency of the transducer (CF) and the −6 dB bandwidth of the transducer (BW), are considered as the output responses of RBFNN model.

The OLHS method [32,33] is employed to sample the data. Compared to other sampling methods, this method ensures that the sample points are not clustered and provides a higher coverage of the overall space, making it efficient in practical applications. To ensure accuracy, the minimum number of samples is determined as S = (N + 1) × (N + 2)/2 + N × 2, where N represents the number of variable factors. To ensure sufficient accuracy, it is appropriate to choose 1.5 to 2 times the minimum number of samples in practical sampling. In order to ensure comprehensive sample point selection and reduce the number of simulations, 50 sample points are selected for the four variable factors within the design space. The performance parameters corresponding to the sample points are calculated by COMSOL Multiphysics v6.0.

The interpolation function of the radial basis function can be expressed as follows:

(2) $$F(x)=\sum_{i=1}^{N}w_{i}\phi_{i}(||X-c_{i}||)$$

where $w_{i}$ represents the weight coefficients, $X\in R^{n}=\left\{x_{1},x_{2,\ldots ,}x_{N}\right\}$, and $c_{i}$ represents the center of the i-th basis function, which is determined by the K-means algorithm [34,35].

It has been demonstrated that a linear combination of Gaussians can approximate any continuous functions with arbitrarily high accuracy. Moreover, neurons with the Gaussian RBF present a very selective response with high activation for patterns close to the radial unit center and tiny activation for the distant pattern. This property reduces the calculated amount and improves the learning rate of the neural network. Therefore, we choose the Gaussian function as the basic function:

(3) $$\phi_{i}(||X-c_{i}||)=\exp\left(-\frac{||X-c_{i}|{|}^{2}}{2\sigma_{i}^{2}}\right)$$

(4) $$\sigma_{i}=\frac{d_{\max}}{\sqrt{2M}}$$

where *X* represents the n-dimensional input variables, $c_{i}$ represents the center of the *i*-th basis function, $\sigma_{i}$ represents the radius of the *i*-th basis function, *d*_max_ is the maximum distance between selected center points, and *M* is the number of nodes in the hidden layer.

According to Equations (2) and (3), the approximate model expression of the neural network can be obtained as follows:

(5) $$Y=\exp\left(-\frac{||X-c_{i}|{|}^{2}}{2\sigma_{i}^{2}}\right)W$$

where *W* represents the optimal weight matrix between the hidden layer and the output layer of the RBFNN, which is trained by the Gradient descent algorithm [34,35].

#### 3.1.2. Error Analysis

**Figure 4:**
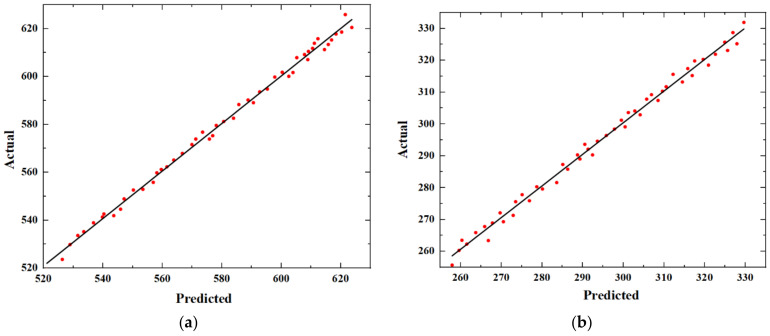

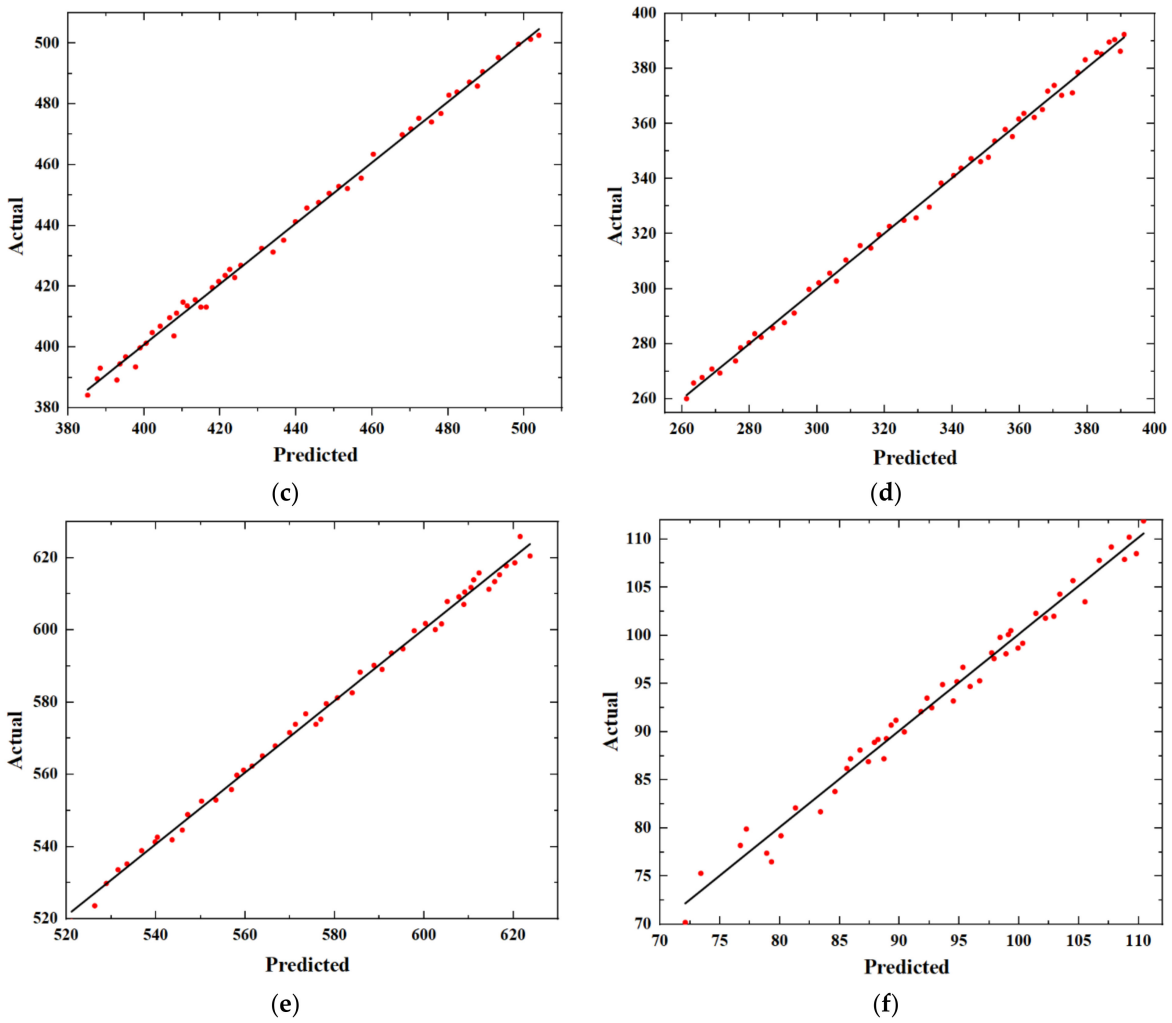
Linear regression plot: (**a**) BG_up1_; (**b**) BG_down1_; (**c**) BG_up2_; (**d**) BG_down2_; (**e**) CF; (**f**) BW.

To assess the accuracy of the approximate model, a linear regression plot is used to compare the predicted values from the RBFNN with the corresponding finite element simulation values. Cross-validation is performed with 50 sample points, and the results are shown in Figure 4, where the horizontal coordinates are the approximate model predictions and the vertical coordinates are the finite element simulation values. From the figures, it can be concluded that the finite element simulation values agree well with the predicted values of the radial basis neural network.

To further assess the reliability of the approximate model established using the RBFNN, this study performs error analysis using three evaluation metrics: normalized root mean square error (*NRMSE*), normalized maximum absolute error (*NMAE*), and reliability indicators ($R^{2}$). The calculations for these metrics are expressed as follows:

(6) $$NRMSE=\sqrt{\frac{\sum_{i=1}^{n}{\left(y_{i}-{\hat{y}}_{i}\right)}^{2}}{\sum_{i=1}^{n}{\left(y_{i}-\bar{y}\right)}^{2}}}$$

(7) $$NMAE=\frac{\max|y_{i}-{\hat{y}}_{i}|}{\sqrt{\frac{1}{n}\sum_{i=1}^{n}\left(y_{i}-{\hat{y}}_{i}\right)}}$$

(8) $$R^{2}=\frac{\sum_{i=1}^{n}{\left({\hat{y}}_{i}-\bar{y}\right)}^{2}}{\sum_{i=1}^{n}{\left(y_{i}-\bar{y}\right)}^{2}}$$

where $y_{i}$ represents the true values obtained from simulations, ${\hat{y}}_{i}$ represents the predicted values from the approximate model, $\bar{y}$ represents the mean value of the true values, and *n* represents the number of experiments or simulations conducted.

The errors of the approximate model obtained by calculating according to Equations (6)–(8) are shown in Table 4. It can be observed that the errors of the approximate model are within an acceptable range.

Based on the linear regression plots and the related accuracy evaluation metrics, it can be concluded that the approximate model obtained using RBFNN can accurately predict the nonlinear relationship between the performance parameters and structural parameters of the phononic crystal air-coupled ultrasonic transducer. Therefore, this model can be used as a substitute for COMSOL simulation results in subsequent optimization designs.

### 3.2. Optimization Design Strategy Based on the NSGA-II Algorithm

**Figure 5:**
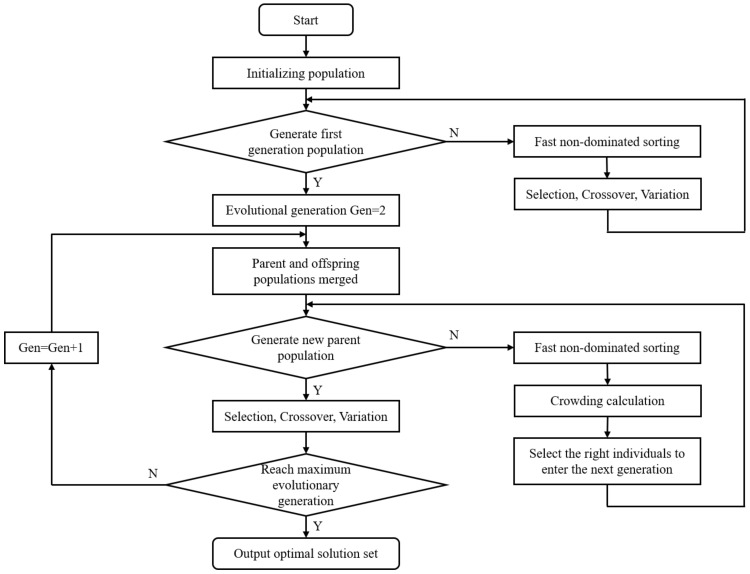
NSGA-II algorithm optimization process.

Due to the large number of optimization variables and objectives, the optimization design problem of the phononic crystal air-coupled ultrasonic transducer can be formulated as a constrained multi-objective optimization problem. The NSGA algorithm exhibits strong search capability for optimal solutions in multi-objective optimization problems and demonstrates good robustness. NSGA-II algorithm, an improved version of NSGA, introduces a crowding distance comparison operator and incorporates an elitist strategy, which expands the sampling space and reduces the computational complexity of non-dominated sorting. As a result, NSGA-II has replaced NSGA as the mainstream algorithm for multi-objective optimization. NSGA-II algorithm is based on the Pareto solution set for solving multi-objective optimization problems, and its main process is illustrated in Figure 5.

In this paper, the objective of the optimization design is to obtain a phononic crystal air-coupled wideband transducer with a center frequency; $CF_{des}=450\;\mathrm{kHz}$. Furthermore, the center frequency of the transducer should be within the bandgap of the 1–3 piezoelectric composites and the acoustic impedance gradient matching layer, and the bandgap widths of both materials should be maximized as much as possible. The nonlinear relationship between the structural parameters and the output responses is predicted by the RBFNN in Equation (5). Therefore, the mathematical expression of the aforementioned optimization model can be written as follows:

(9) $$\left\{\begin{array}{l} \min\left\{\frac{CF-CF_{des}}{CF_{\max}-CF_{\min}},\frac{1}{BG_{up1}-BG_{down1}},\frac{1}{BG_{up2}-BG_{down2}},\frac{1}{BW}\right\}\\ s.t\left\{\begin{array}{l} 2.5\;\mathrm{mm}\leq X_{1}\leq 3\;\mathrm{mm}\\ 0.85\;\mathrm{mm}\leq X_{2}\leq 1.15\;\mathrm{mm}\\ 2.5\;\mathrm{mm}\leq X_{3}\leq 3.5\;\mathrm{mm}\\ 2\;\mathrm{mm}\leq X_{4}\leq 3\;\mathrm{mm} \end{array}\right\} \end{array}\right\}$$

where *CF_des_* represents the desired center frequency of the transducer, *CF*_max_ represents the maximum calculated center frequency of the transducer, and *CF*_min_ represents the minimum calculated center frequency of the transducer.

Based on the obtained optimization model, iterative calculations are performed using the NSGA-II algorithm. The parameter settings for the algorithm are shown in Table 5. Finally, we obtain the final optimized results that satisfy the constraint conditions. After rounding treatment, the structural parameters are shown in Table 6.

## 4. Verification

### 4.1. Simulation Verification

**Figure 6:**
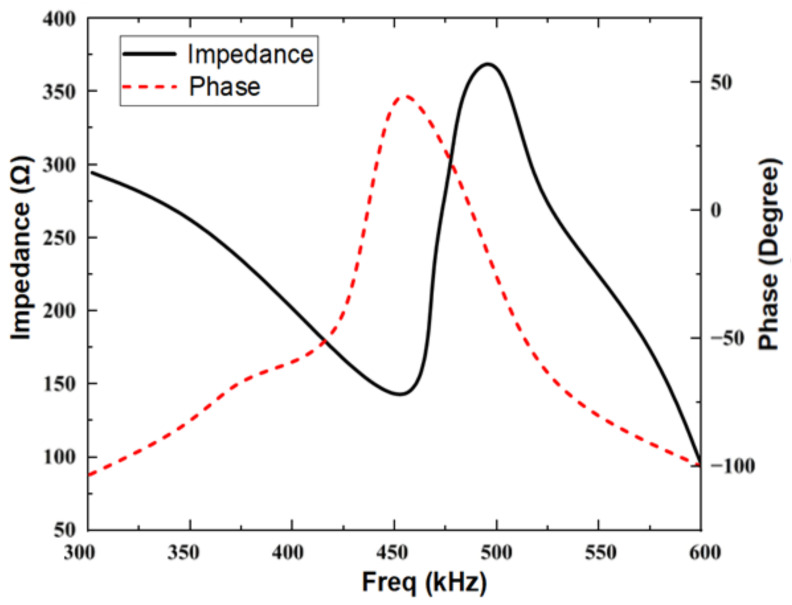
Impedance-phase curve of the transducer.

**Figure 7:**
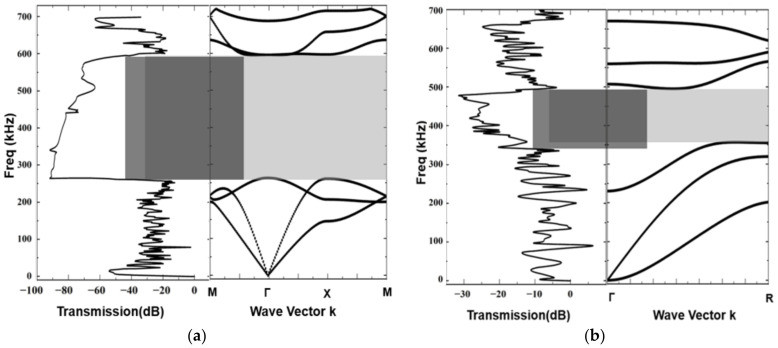
Transmission characteristic curve and Energy band structure after optimization: (**a**) 1–3 piezoelectric composites; (**b**) acoustic impedance gradient matching layer.

Based on the optimized design parameters, the impedance-phase curve of the transducer (Figure 6) is simulated using COMSOL Multiphysics V6.0. The resonant frequency of the transducer is approximately 455.2 kHz, which is in close agreement with the design requirement. From the energy band structure and transmission characteristic curve of the optimized 1–3 piezoelectric composites and acoustic impedance gradient matching layer (Figure 7), it can be observed that the bandgap range of the piezoelectric composite is 262 kHz to 583 kHz, and the bandgap range of the acoustic impedance gradient matching layer is 337 kHz to 498 kHz. The operating frequency of the transducer falls within these band gaps. Moreover, compared to the pre-optimization, the bandgap widths have increased by 16 kHz and 13.5 kHz, respectively.

**Figure 8:**
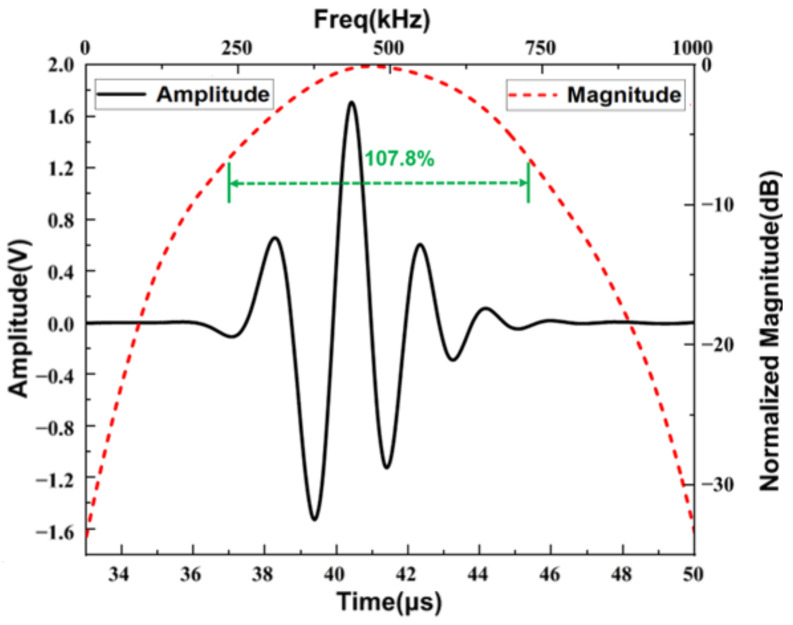
Pulse-echo simulation result.

Figure 8 shows the time domain and frequency domain plots of the echo signal obtained from the pulse echo analysis of the transducer model. In order to compare the difference between the operating performance of each transducer model, we investigate the −6 dB bandwidth (*BW*), which can be defined using the following equations:

(10) $$BW=\frac{2\left(f_{H}-f_{L}\right)}{f_{H}+f_{L}}$$

where $f_{H}$ and $f_{L}$ are the frequency points corresponding to the 6 dB lower of the maximum value of fast Fourier transform.

From the time and frequency domain analysis of the echo signal, it can be calculated that the BW of the optimized transducer is about 107.8%, which represents an improvement of 11.5% compared to the pre-optimized. Therefore, the proposed optimization design strategy for the phononic crystal air-coupled ultrasound transducer is effective and feasible.

### 4.2. Experimental Verification

**Figure 9:**
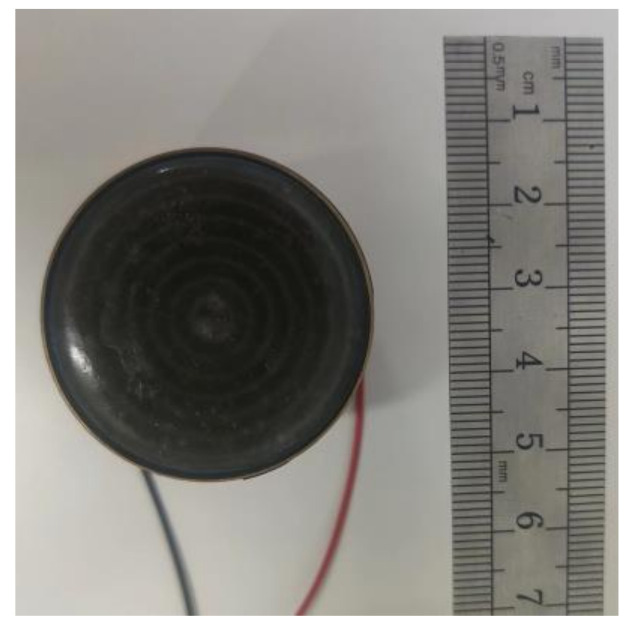
Optimized transducer sample.

To further verify the effectiveness of the optimization design strategy, a transducer sample (Figure 9) is fabricated based on the optimized results. In practical testing, to prevent interference from acoustic wave reflections generated by the piezoelectric material during operation, polyurethane material is chosen as the backing material for the transducer to absorb the reflected acoustic waves. The matching layer, the piezoelectric material and the backing are bonded to each other using epoxy resin.

**Figure 10:**
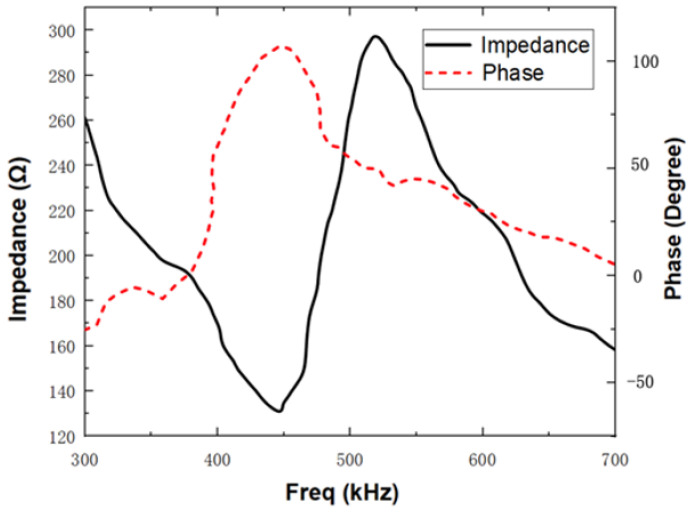
Impedance-phase curve of optimized transducer sample.

First, the fabricated transducer was subjected to impedance testing using the HIOKI-IM3570 Precision Impedance Analyzer to determine its resonant frequency. Due to variations in the driving frequency of the transducers, there are corresponding differences in the internal currents generated. When the driving frequency matches the mechanical resonant frequency of the transducer, the internal current reaches a peak. Therefore, the actual resonant frequency of the transducer can be obtained through impedance measurements. During the test, the impedance analyzer was used to sweep the connected transducer samples, resulting in impedance-phase curves for each corresponding frequency. The test results are shown in Figure 10, and it can be observed that the resonant frequency of the fabricated transducer is 445 kHz.

**Figure 11:**
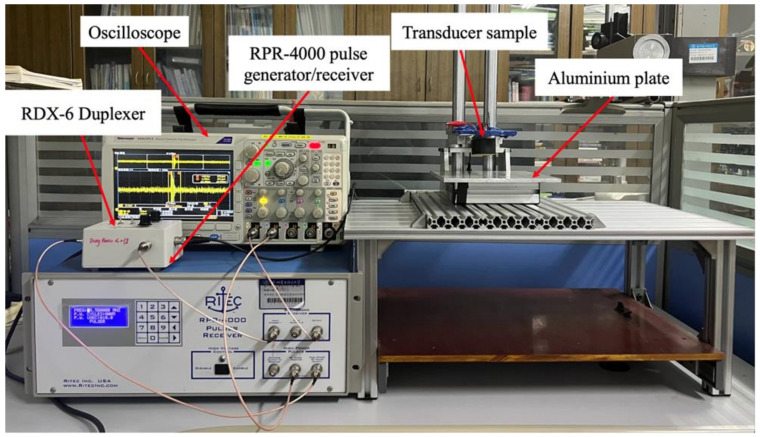
Pulse-echo test platform.

**Figure 12:**
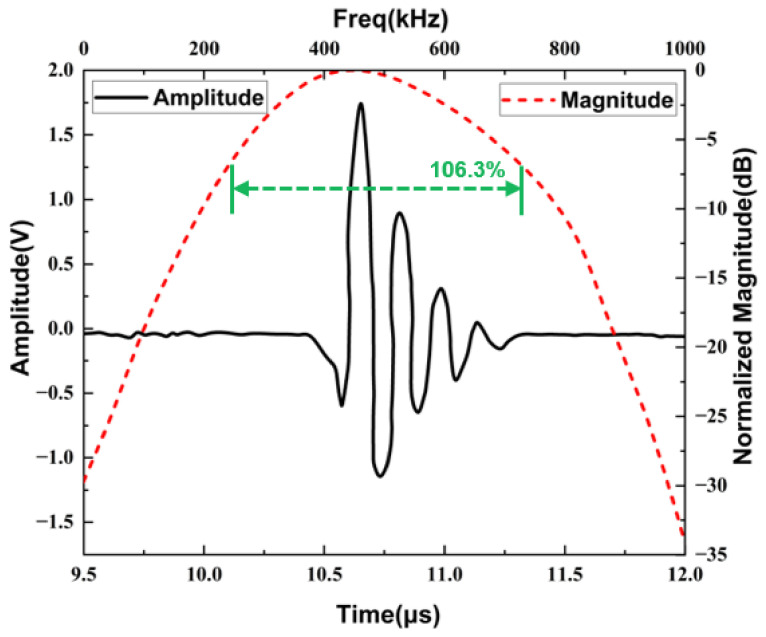
Pulse-echo test result.

After determining the resonant frequency of the transducer sample, pulse-echo analysis was performed to test its operational performance. A pulse-echo test platform (Figure 11) was assembled for this purpose. First, the RITEC RPR-4000 high-power pulse generator/receiver is used to generate the excitation signal, which is then transmitted to the ultrasonic transducer samples through the RDX-6 duplexer module. The transducer emits ultrasonic waves under the action of the excitation signal, and these waves travel through the air, reach the aluminum plate, and produce echoes. The echoes are received by the transducer again through the duplexer, generating echo signals that are displayed on an oscilloscope. During the experiment, it is essential to keep the transducer surface parallel to the surface of the aluminum plate, and the distance between the transducer and the aluminum plate should be 20 mm. Under these conditions, the received echo signal is in the time domain. To obtain the corresponding frequency spectrum, the time-domain waveform is subjected to fast Fourier transform. To avoid experimental interference, it is preferable to use narrowband high-power signals to excite the transducer with a relatively single mode. In this study, a 5-cycle sine wave with rectangular window modulation provided by the RPR-4000 system, is chosen as the excitation signal. The excitation frequency corresponds to the resonant frequency of each transducer obtained from the impedance analyzer, with an emission voltage of 10 V_pp_. The test result (Figure 12) shows that the −6 dB bandwidth of the transducer is 106.3%. The test result closely aligns with the simulated result, further validating the effectiveness of the optimization design strategy.

## 5. Discussion

The obtained results from intelligent optimization, finite element simulation, and experimental testing demonstrate consistency. However, it is observed that the measured resonant frequency of the transducer is slightly lower compared to the simulated value. The main reason for the discrepancy is that during the experiment, impedance testing was conducted on the transducer, while in the simulation, only the impedance analysis of the piezoelectric composite material was conducted, neglecting the effects of the backing layer and matching layer. The backing layer primarily serves to absorb acoustic waves emitted by the piezoelectric composite material toward the backside, thereby reducing reflection. However, in the experiment, it is not possible to completely absorb the acoustic waves, which may lead to a slight deviation in the resonant frequency. The backing layer and excessive epoxy resin in bonding the parts increase the mass and stiffness of the transducer, which can also lead to a lower resonant frequency. Furthermore, the thickness of the fabricated piezoelectric material might be larger than the designed thickness due to limitations in processing precision, which could also contribute to this phenomenon. Despite the variation, the experimentally measured resonant frequency still falls within the bandgap frequency range of the piezoelectric composite material and the acoustic impedance gradient-matched layer. The suppression effect on transverse vibration is not significantly weakened. Therefore, the impact of this deviation on the overall performance of the air-coupled ultrasonic transducer is minimal.

The consistency between the finite element simulation and experimental results confirms the improved performance of the optimized transducer, validating the effectiveness of the proposed optimization design strategy for phononic crystal air-coupled ultrasound transducer based on RBFNN and NSGA-II algorithm. However, during the optimization process, we only consider the influence of four factors: the thickness and piezoelectric column radius of 1–3 piezoelectric composite, as well as the thickness and period of the acoustic impedance gradient matched layer, while neglecting the impact of other factors. For example, the backing layer, which primarily serves for sound absorption and damping, prevents acoustic waves from reflecting back to the piezoelectric composite material. The material, structure, and thickness of the backing layer determine the effectiveness of backward sound wave suppression, further affecting the bandwidth of the ultrasonic transducer. Additionally, the relative position of the resonant frequency of the transducer within the bandgap of the piezoelectric composite material and the acoustic impedance gradient matched layer was found to influence the suppression effect of transverse vibration. Moreover, when the filling ratio of the piezoelectric composite is fixed, changes in the lattice constant can alter the bandgap width and position, consequently affecting the suppression effect of transverse vibration. Therefore, the lattice constant of the piezoelectric composite is another potential influencing factor. Certainly, considering more influencing factors will bring forth new potential challenges. Firstly, it increases the complexity of the optimization problem, and the complexity and workload of training the RBFNN model and implementing the NSGA-II algorithm will significantly escalate. Additionally, during the optimization process, certain trade-offs or compromises may be required. For instance, in pursuit of placing the resonant frequency in the middle of the bandgap, it might be necessary to sacrifice a portion of the bandgap width.

## 6. Conclusions

In this study, based on the RBFNN model and NSGA-II algorithm, an optimization design strategy for the piezoelectric layer and impedance gradient matching layer of phononic crystal air-coupled ultrasound transducer is proposed to further improve its performance. Firstly, by combining the OLHS method with simulation data obtained from COMSOL software, a RBFNN model is trained to describe the nonlinear relationship between the design parameters and performance parameters of the transducer. The accuracy of the approximate model is verified through error analysis using linear regression plots and statistical methods. Then, the optimal design parameters of the transducer are determined based on the RBFNN model and NSGA-II algorithm. Furthermore, the effectiveness of the proposed optimization design strategy is verified through simulations and experiments. After optimization, the resonant frequency of the transducer falls within the bandgap range of both the piezoelectric composite material and the acoustic impedance gradient matching layer. Additionally, the bandgap width of the piezoelectric composite material and the acoustic impedance gradient matching layer is increased by 16 kHz and 13.5 kHz, respectively, and the −6 dB bandwidth of the transducer is improved by 11.5%. The optimized phononic crystal air-coupled ultrasound transducer holds great potential for applications in non-destructive testing.

## Figures and Tables

**Table 1:** 1–3 piezoelectric composite material parameters.

Material	Epoxy Resin	PZT-5A
Density $\rho /\mathrm{kg}\cdot m^{-3}$	1063	7750
Young’s modulus $E/10^{9}\;\mathrm{Pa}$	3.5	56
Poisson’s ratio	0.38	0.36

**Table 2:** Acoustic impedance gradient matching layer material parameters.

Materials	Density $\rho /\mathrm{kg}\cdot m^{-3}$	Young’s Modulus $E/10^{9}\;\mathrm{Pa}$	Poisson’s Ratio	Acoustic Impedance/MRayl
Epoxy resin	1063	3.5	0.38	2.7
AlSi10Mg	2660	75	0.3	16.7

**Table 3:** Design variables.

	Variable Factor	Design Parameter	Initial Value (mm)	Lower Limit (mm)	Upper Limit (mm)
1–3 piezoelectric composites	X_1_	thickness (T)	2.5	2	3
	X_2_	column radius (r)	1	0.85	1.15
gradient matching layer	X_3_	thickness	3	2.5	3.5
	X_4_	period (Λ)	2.5	2	3

**Table 4:** RBFNN model error analysis.

Evaluation Metrics	NRMSE	NMAE	$R^{2}$
Acceptable range	≤0.2	≤0.3	≥0.9
BG_up1_	0.072	0.093	0.989
BG_down1_	0.026	0.062	0.995
BG_up2_	0.015	0.042	0.997
BG_down2_	0.072	0.093	0.989
CF	0.067	0.071	0.991
BW	0.096	0.098	0.985

**Table 5:** NSGA-II algorithm parameters.

Parameter	Iteration Number	Population Size	Crossover Probability	Variation Probability
Optimization Result	1000	1000	0.9	0.1

**Table 6:** Optimization result parameters.

Variable Factor	X_1_ (mm)	X_2_ (mm)	X_3_ (mm)	X_4_ (mm)
Optimization Result	2.58	0.85	2.85	2.3

## Data Availability

The data presented in this study are available on request from the corresponding author. The data are not publicly available due to privacy.
